# The Art of Chromosome Capture: Kinetochore Structures Across Evolution.

**DOI:** 10.1063/4.0000917

**Published:** 2025-10-27

**Authors:** Stanislau Yatskevich

**Affiliations:** Genentech, South San Francisco, CA, USA

## Abstract

Kinetochores are essential macromolecular machines that anchor chromosomes to the mitotic spindle, ensuring accurate chromosome segregation during cell division. To perform this task, kinetochores must assemble at centromeres, forge stable chromatin-spindle connections, and withstand the relentless forces applied during mitosis without losing their grip on DNA.

Structural studies have unveiled both strikingly conserved and remarkably divergent principles guiding kinetochore architecture. From the point- centromeres of budding yeast to the regional centromeres of humans and to the holocentric chromosomes of insects, a unifying theme is that all kinetochores, in their monomeric state, topologically entrap DNA within a central CENP-LN channel. This entrapment acts like a molecular clasp, allowing kinetochores to resist multidirectional spindle forces during mitosis.

However, in a fascinating twist, both yeast and holocentric insects form head-to-head kinetochore dimers, and holocentric species also harness the energy of dimerization to bend DNA into loop-like structures primed for chromosome segregation. Human kinetochores, on the other hand, deploy histone-fold domain proteins (CENP-TWSX) to wrap DNA around themselves, forming a similar loop but—so far—only in monomeric form. These insights reveal a stunning example of evolutionary convergence: despite their diversity, kinetochores across eukaryotes rely on sequence-independent, topological DNA entrapment as a fundamental mechanism. Yet, species-specific innovations in centromere recognition, dimerization, and DNA bending underscore the remarkable plasticity of these ancient machines.

